# Primary ovarian angiosarcoma in a 12- year -old girl: a case report of an exceptional localization in a context of limited resources country

**DOI:** 10.1186/s12907-017-0056-x

**Published:** 2017-08-24

**Authors:** Tchin Darré, Abdoul-Samadou Aboubakari, Bingo K. N’Bortche, Akila Bassowa, Solange Adani-Ifé, Gado Napo-Koura

**Affiliations:** 1 Department of Pathology, University Teaching Hospital, BP 1515 Lomé, Togo; 2 Department Obstetrics and Gynecology, University Teaching Hospital, Lomé, Togo; 3 Department Obstetrics and Gynecology, University Teaching Hospital, Kara, Togo; 4 Department of Clinical Oncology, University Teaching Hospital of Lomé, Lomé, Togo

**Keywords:** Primary angiosarcoma, Ovary, Childhood, Togo, Sub-Saharan Africa

## Abstract

**Background:**

Ovarian sarcomas represent less than 1% of all ovary cancers and usually are frequent in adults. Primary angiosarcomas are exceptional in the ovaries within children.

**Case presentation:**

We reported a case of primary ovarian angiosarcoma in a 12-year-old girl in a resource-constrained context. Immunohistochemistry study showed the positivity of CD34, CD31, factor VIII, while S100 was negative. The diagnosis of primary non-metastatic angiosarcoma was retained. She was unable to undergo the CWS-2002P chemotherapy since her parents could not afford it.

**Conclusion:**

This case report described a rare type of a primary ovarian angiosarcoma within a child, diagnosed in a low-income country in a laboratory with limited resources.

## Background

Ovarian sarcomas are very rare and represent less than 1% of all malignant ovarian tumors [1]. Angiosarcomas (AS) are rare vascular cancers, 2–3% of soft tissue sarcomas with high aggressively and frequent metastasis [2, 3]. AS are prevalent in the adult subjects and ovarian localization is rarely described in the literature. Like most malignant tumors, the diagnosis of certainty is done by the conventional histology technique and the immunohistochemical testing with the positivity of specific markers of the vascular endothelium [4]. We report a case of primary angiosarcoma of the ovary in a 12-year-old girl in a resource-constrained context.

## Case presentation

A 12-year-old female had consulted at the Department of Gynecology for pelvic pain for 22 days which was intense in the evening at lying. She had a polyuria for 10 days and occurred over the night where she woke up 7 times on average to urinate. There was no notion of traumatism. There was no medical and surgical history. There was no concept of sexually transmitted infections. The socio-economic level was considered average; she had never smoked and did not drink alcohol. She had normal pubertal development with chest changes and pubic hair.

Clinical examination displayed a temperature of temperature at 37 °C, a weight of 46 kg for a size of 1.34 m, BMI was 25,62Kg/m^2^. The blood pressure was 110/60mmhg. The general condition was good. The patient suffered from pain in the right pelvic region triggered by palpation, and thus, limiting the movement of the right hip. Palpable adenopathy was absent. The remaining physical examination was normal. The biological examination was normal. The abdominal ultrasound showed a solid mass that occupied the right ovary. The biological check-up was in normal. Alpha-fetoprotein and β-hCG were normal. Ultrasound of the abdomen showed a solid mass that occupied the right ovary.

The patient underwent an ovariectomy. The macroscopic analysis revealed that the ovariectomy was grayish, irregular and elastic and measured 17cmx14cmx9cm. The ovarian sections were heterogeneous, including red areas and hemorrhagic territories. The normal ovarian tissue was not identified. The Fallopian tube was normal. The contralateral adnexa and the uterine body were normal. The peritoneum was apparently normal and the peritoneal cavity contained approximately 1000 mL of blood ascetic fluid.

**Fig. 1:**
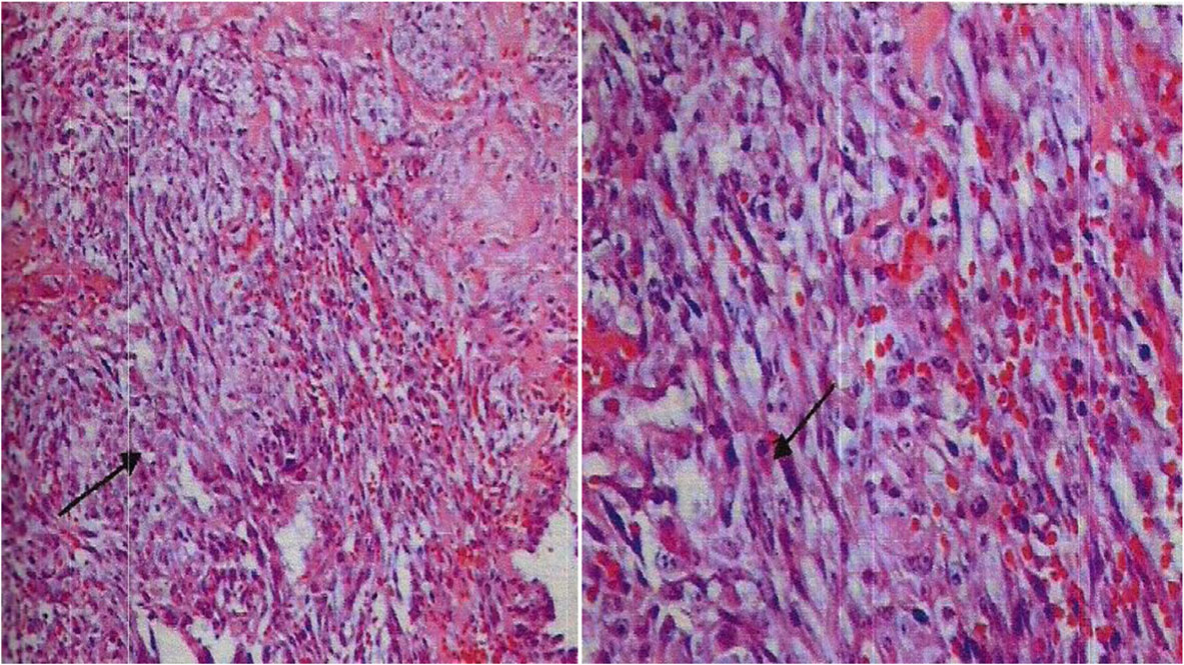
Angiosarcoma undifferentiated. Note the presence of anarchic vascular elements not very differentiated with mitoses (arrow) (HE, G X 200 and 400)

The sections of the ovarian tumor were made and ten blocks of paraffin were made. Conventional histology showed a malignant vascular tumor proliferation made of many very small vascular luminesces with endothelial cells exhibiting nuclear atypia and mitoses. The necrosis was present. As diagnostic hypotheses, we primarily reported angiosarcoma followed by hemangiopericytoma (Fig. 1). Three blocks of paraffin embedded ovarian tissue were sent to a well-equipped laboratory in France for immunohistochemistry analysis to confirm the diagnosis.

**Fig. 2:**
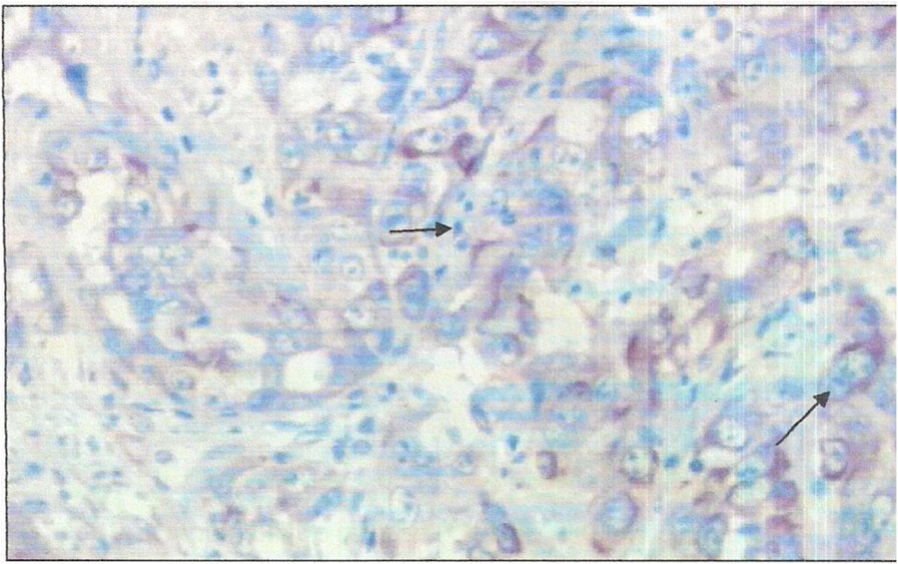
Factor VIII evidence in immunohistochemistry (arrow) (G X 400)

**Fig. 3:**
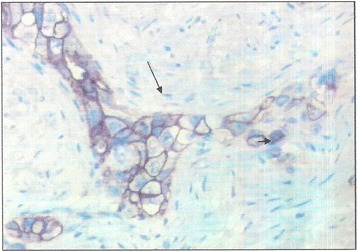
Evidence for CD34 tumor markers in immunohistochemistry (arrow) (G X 400)

**Fig. 4:**
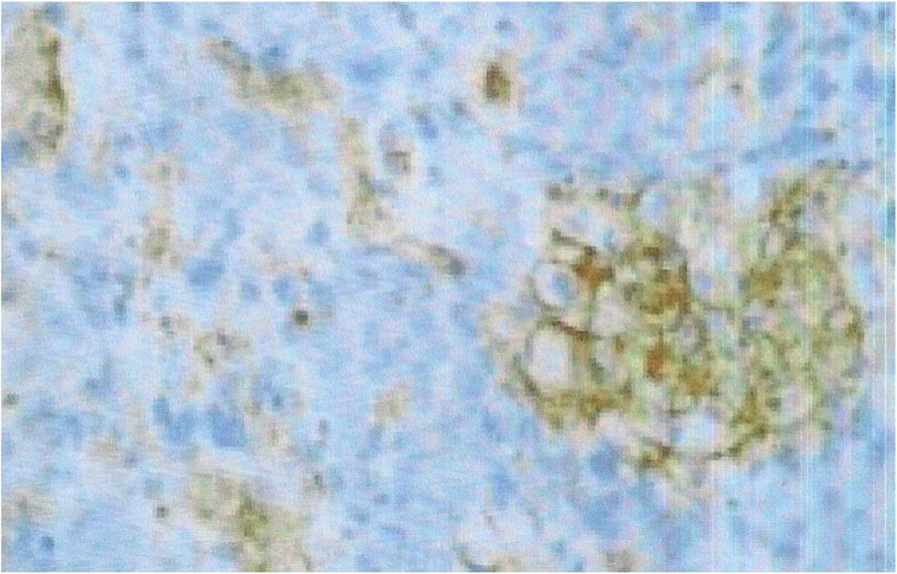
Evidence for CD34 tumor markers in immunohistochemistry (G X 400)

The result of immunohistochemistry analysis was CD31+, CD34+, and Factor VIII+ (Figs. 2, 3 and 4). The tumor cells were SMA-, desmin-, melanin A-, and S-100-. The diagnosis of grade 2 angiosarcoma (FNCLCC) was confirmed after a multidisciplinary consultation. The patient was eligible for chemotherapy according to the CWS-2002P protocol. However, the patient’s family had a financial issue.

## Discussion

Angiosarcomas are uncommon vascular cancers, accounting for 1–2% of soft tissue sarcomas with high aggressive and metastatic potential [1, 3]. Angiosarcoma in children is of 0.3% of all pediatric sarcomas. Pediatric angiosarcomas have been reported in the soft tissues of head and neck, trunk and extremities, bone, heart, liver spleen and abdomen or pelvis [5]. In the Polish Paediatric Rare Tumours Study, onne of the 10 patients with angiosarcoma had bladder as the primary site [6]. Of the 14 patients reported by Deyrup et al., one had angiosarcoma arising in the pelvis [7]. Only 3 cases of ovarian angiosarcoma were reported in patients younger than 21 years, and all died within a year of diagnosis [8].

Angiosarcoma has been identified as the second most frequent sarcoma in germ cell tumors with sarcomatous components after rhabdomyosarcoma [9]. This raised the hypothesis that the original tumor of our patient was a ovary teratoma following a malignant transformation. However, the histopathologic examination of the ovary did not reveal teratoma or the most probable primary site of the angiosarcoma were the ovary. Surgical and pathologic evaluation of this patient’s tumor led to the conclusion that the most likely primary site angiosarcoma was the ovary.

The histopathological diagnosis of angiosarcoma is challenging. Angiosarcoma may be confused with vascular tumors of intermediate malignancy (e.g., epithelioid and spindle cell hemangioendotheliomas, histioid hemangioma, and malignant endovascular papillary angioendothelioma) [1, 3, 4]. Hemangioperycitoma is a designation for vascular tumors that are a histological intermediate in appearance between a hemangioma and a conventional angiosarcoma. Anaplasia is prominent in these tumors comprised of groups of irregular vascular elements lined by immature endothelial cells. The International Society for the Study of Vascular Anomalies published the most up to date classification of vascular lesions in 2014, distinguishing malformations from tumors [10]. Angiosarcoma is classified as a malignant tumor, along with epithelioide hemangioperycitoma. Angiosarcoma is characterized by pleomorphic endothelial cells that can be rounded, polygonanal, or spindled [11]. They have prominent nucleoli, abundant cytoplasm, and sometimes contain intracytoplasmic clusters of erythrocytes [12].

These abnormal endothelial cells typically form vascular sinusoids that infiltrate the surrounding tissue. The vessels may be lined by a single or multiple layers of malignant cells, and thus creating an irregular network of vascular lumina. The vascular spaces may be difficult to recognize due to the solid appearance of the tumor in case of poorly differentiated angiosarcoma [12]. Mitotic activity, hemorrhagic areas, and necrosis are relevant. The differential diagnostics of this atypical angiosarcoma may be poorly differentiated Kaposi’s sarcoma, fibrosarcoma, and leiomyosarcoma [1, 3, 12].

Immunohistochemistry (IHC) is relevant for the certainty of angiosarcoma diagnosis. This technique carried out in a Laboratory of Anatomic Pathologyin France revealed the positivity of specific markers of the angiosarcoma such as the factor 8, CD31, and CD34. It is clearly shown that IHC is an essential technique in the diagnosis of undifferentiated angiosarcomas [13]. CD34 may be focally positive or negative in some cases. CD34 may be only focally positive, or in some cases negative. CD31 is more sensitive than CD34, although its sensitivity decreases with more solid and undifferentiated angiosarcoma [4, 13]. The same occurs with Factor 8 because it is a marker of differentiated endothelial cells. The negativity of S-100 differentiates angiosarcoma from melanoma. Vimentin is positive to variable degrees in epithelioid angiosarcomas, making them difficult to differentiate from carcinomas. However, the endothelial markers previously discussed are negative in carcinomas [13, 14].

Angiosarcomas badly respond to chemotherapy and radiotherapy. Surgery is the key treatment of the early form of this cancer although there is common recurrence [15]. An optimal adjuvant therapy is unknown, but patients receive both single agent therapy as well as multiple-drug regimens. Because local or distant recurrence is possible despite adequate excision, adjuvant therapy may be of benefit [15].

The prognosis of angiosarcoma in general is dismal, with a 5-year survival rate lower than 30% for non-metastatic angiosarcoma, and an over-all survival of 8 months for the metastatic stage [16]. Although a longer follow-up is indeed necessary, our patient has had difficulties in taking care of the therapist due to the lack of financial means of her family. As our country does not yet have a real policy for the management of cancerous pathologies, it is often up to the family to pay their patients’ treatment costs. In addition, existing health insurance covers only public servants. All these difficulties may complicate the management of the patient.

## Conclusion

Angiosarcomas are extremely rare vascular tumors in children and ovarian localization is exceptional with high mortality. Its diagnosis must be sought in tumors with a rich vascular network, necrosis and significant mitotic activity. This observation also shows the diagnostic difficulties encountered by pathology laboratories under equipped in developing countries and poses the problem of therapeutic management in our country.

## Abbreviations

Gx: Magnification
HE: Haematein eosin
